# Crying wolf: concluding that wolves were not restored

**DOI:** 10.1098/rsbl.2008.0440

**Published:** 2008-10-14

**Authors:** L. David Mech

**Affiliations:** U.S. Geological Survey, Northern Prairie Wildlife Research Center8711—37th Street Southeast, Jamestown, ND 58401-7317, USA

In 2007, the USA removed (delisted) the grey wolf (*Canis lupus*) in the upper Midwest from the Endangered Species List. After 35 years of being considered endangered, these wolves had increased from 750 in 38 400 km^2^ of Minnesota (Fuller *et al*. 1992) to over 4100 inhabiting 110 000 km^2^ of Minnesota, Wisconsin, and Michigan. Conservationists celebrated this event as a most significant success. Suddenly, however, this milestone was challenged by Leonard & Wayne (2007) based on preliminary genetic findings that wolves of the upper Midwest 100 years ago were different from 69 per cent of the current population. There are, however, both technical and logical problems with this challenge.

Leonard & Wayne (2007) examined the mitochondrial DNA of 17 century-old specimens from eastern Wisconsin, Michigan, and southern Ontario. Comparing them with 68 late-1980s specimens from across northern Minnesota (figure 1), they concluded that the haplotypes of the old specimens were found in only 31 per cent of the recent specimens. Apparently then assuming that their current sample was representative of the 4100 recovered wolves, these authors concluded that the current population should not be considered recovered and thus should not have been delisted.

However, lack of representativeness is a major problem with the recent Minnesota sample (Lehman *et al*. 1991). First, because wolves of similar genotypes tend to segregate (Geffen *et al*. 2004), a non-spatially representative sample could miss haplotypes similar to the older specimens. Thus more than 31 per cent of the present wolves could possess the older haplotype.

Second, in the current range of the recovered wolves, the old specimens from Wisconsin, Michigan, and Ontario can be fairly compared with only two types of samples: (i) current wolves from the same area and (ii) century-old specimens from Minnesota, excluding extreme northeastern Minnesota. Leonard & Wayne (2007) made neither comparison. This is important because the few known founders of the recolonized Wisconsin and Michigan population originated in northeastern rather than northwestern Minnesota (Mech *et al*. 1995). If most Wisconsin–Michigan founders were from northeastern Minnesota, then the failure to compare the old specimens with the current Michigan–Wisconsin specimens undermines the argument that the recovered population includes too few haplotypes present a century ago.

Third, the closest old specimen was taken 360 km from extreme northeastern Minnesota where wolves differ from those farther west. The wolves in extreme northeastern Minnesota, primarily Cook County, are significantly lighter weight than those in northwestern Minnesota (Mech & Paul 2008). Differences between these two wolf types have long been recognized; Young & Goldman (1944) considered the northwestern Minnesota wolf *Canis lupus nubilus* and those in northeastern Minnesota, *Canis lupus lycaon*. This fact makes it imperative to include extreme northeastern Minnesota wolves in any sample representative of Minnesota. The Lehman *et al*. (1991) sample contained only one such specimen.

Fourth, the conclusion that the current 4100 upper Midwest wolves should not have been delisted ignores the fact that current legal criteria for their delisting have been achieved. This wolf population is protected by state laws, except for minor numbers taken for livestock-depredation control, and populations in all three states continue to increase. Furthermore, the federal government must monitor wolf status under state and tribal management there for five years after delisting, and even after that could relist the wolves quickly if necessary.

Even more important is the fact that, biologically, the number of wolves in the Midwest has reached a level that is highly unlikely to be reduced by any legal means. The original endangerment was caused primarily by poisoning and aerial hunting, which are no longer legal. To reduce the current population would require annual removal of 1500–2500 wolves (Fuller *et al*. 2003).

The Leonard & Wayne (2007) study raised interesting genetic issues, and further comparisons between their century-old specimens, other current specimens, older Minnesota specimens and representative samples from the past and current distribution would be informative. The authors themselves state that ‘nuclear DNA analyses are needed to further confirm this conclusion’ (Leonard & Wayne 2007, p. 3). However, if one relies solely on the data presented by Leonard & Wayne (2007), the conclusion that the upper Midwest wolves should be returned to the Endangered Species List is plainly a case of crying wolf.

## Figures and Tables

**Figure 1:**
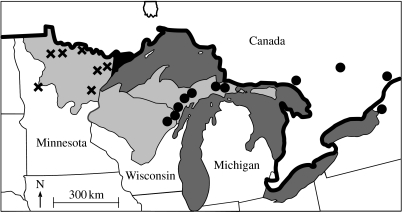
The upper Midwest of the USA with current wolf range (light grey area), locations of specimens examined by Leonard & Wayne (2007; black circles—apparently some represent more than one specimen), Minnesota counties (crosses) where current specimens originate (Lehman *et al*. 1991) and Cook Co., MN (black area in northeastern Minnesota).

## References

[bib1] Fuller T.K., Berg W.E., Radde G.L., Lenarz M.S., Joselyn G.B. (1992). A history and current estimate of wolf distribution and numbers in Minnesota. Wildl. Soc. Bull.

[bib2] Fuller T.K., Mech L.D., Fitts-Cochran J., Mech L.D., Boitani L. (2003). Population dynamics. Wolves: behavior, ecology, and conservation.

[bib3] Geffen E., Anderson M.J., Wayne R.K. (2004). Climate and habitat barriers to dispersal in the highly mobile grey wolf. Mol. Ecol.

[bib4] Lehman N., Eisenhawer A., Hansen K., Mech L.D., Peterson R.O., Gogan P.J.P., Wayne R.K. (1991). Introgression of coyote mitochondrial DNA into sympatric North American gray wolf populations. Evolution.

[bib5] Leonard J.A., Wayne R.K. (2007). Native Great Lakes wolves were not restored. Biol. Lett.

[bib6] Mech L.D., Paul W.J. (2008). Wolf body mass cline across Minnesota related to taxonomy?. Can. J. Zool.

[bib7] Mech L.D., Fritts S.H., Wagner D. (1995). Minnesota wolf dispersal to Wisconsin and Michigan. Am. Midl. Nat.

[bib8] Young S.P., Goldman E.A. (1944). The wolves of North America.

